# Distribution and extent of suitable habitats of Ruspoli’s Turaco (*Tauraco ruspolii)* and White-cheeked Turaco (*Tauraco leucotis*) under a changing climate in Ethiopia

**DOI:** 10.1186/s12862-024-02245-y

**Published:** 2024-06-21

**Authors:** Mulatu Ayenew Aligaz, Chala Adugna Kufa, Ahmed Seid Ahmed, Hailu Tilahun Argaw, Misganaw Tamrat, Mesele Yihune, Anagaw Atickem, Afework Bekele, Bezawork Afework Bogale

**Affiliations:** 1 Department of Biology, Debre Markos University, P.O. Box, 269, Debre Markos, Ethiopia; 2 Department of Biology, Natural and Computational Sciences, Woldia University, P.O. Box, 400, Woldia, Ethiopia; 3 Department of Biology, Hawassa University, P. O. Box 05, Hawassa, Ethiopia; 4 Department of Wildlife and Ecotourism Management, Wolkite University, P.O. Box. 07, Wolkite, Ethiopia; 5 Department of Zoological Sciences, Addis Ababa University, P.O. Box. 1176, Addis Ababa, Ethiopia

**Keywords:** Climate change, Endemic species, Habitat suitability overlap, Maximum entropy, Species distribution modeling, Turaco species

## Abstract

**Background:**

Understanding the distribution pattern of species and their suitable habitat is key to focus conservation efforts. Climate change has had notable impact on the distribution and extent of suitable habitats, and the long-term survival of various species. We aim to determine the distribution and extent of suitable habitats for *Tauraco ruspolii* and *T. leucotis* in Ethiopia and predict their range in the 2050s and 2070s using MaxEnt algorithm. We used 25 and 29 rarified occurrence points for *T. ruspolii* and *T. leucotis*, respectively, and 13 environmental variables. Three regularization multipliers and two cut-off thresholds were used to map the potential suitable habitats for each species under current and future climates. Maps were assembled from these techniques to produce final composite tertiary maps and investigated the habitat suitability overlap between the two species using the UNION tool in the geographical information system.

**Result:**

All model run performances were highly accurate for both species. Precipitation of the driest month and vegetation cover are the most influential variables for the habitat suitability of *T*. *ruspolii*. The habitat suitability of *T. leucotis* is also mainly influenced by mean temperature of the driest quarter and vegetation cover. Under the current climate, the suitable habitat predicted for *T. ruspolii* covered about 24,639.19 km^2^, but its range size change shows a gain and increase by 156.00% and 142.68% in 2050 and 2070, respectively. The *T. leucotis‘s* current suitable habitat ranges about 204,397.62 km², but this is reduced by 40.84% and 68.67% in 2050 and 2070, respectively. Our modeling also showed that there was suitable habitat overlap between them at the margin of their respective habitat types in time series.

**Conclusion:**

We concluded that there is a direct or indirect impact of climate change on the suitable habitat range expansion for *T. ruspolii* and contraction for *T. leucotis* as well as overlapping of these turaco species in different regions of Ethiopia. Therefore, understanding the distribution of current and future suitable habitats of the two turaco species can provide valuable information to implement conservation practices for the species and the regions as well.

**Supplementary Information:**

The online version contains supplementary material available at 10.1186/s12862-024-02245-y.

## Introduction

The distribution pattern of species and the availability of their suitable habitats were mainly affected by climate change which is driven by anthropogenic pressures at a global scale in the current Anthropocene Epoch [1–4]. Recently, there has been an increasing interest in modeling and mapping the habitat suitability of species including birds to prioritize conservation areas and predict the possible changes of their suitable habitats due to climate change [5–8].

Bird species, like other species, either adapt to the accelerating climate change, shift out of their natural range (i.e., loss or gain), or become extinct [5, 6, 8]. Shifting is usually altitudinal or latitudinal [9–11]. Restricted range birds are more vulnerable to extinction when they experience climate change due to loss of the suitable habitats [6, 12]. Globally, an increase of temperature by 1ºC is projected to have a non-linear increase in bird extinction by 100–500 species in future climatic conditions [6]. This is more severe for forest-specialist birds in the Afro-tropic biogeographic realm since they require specific ecological conditions [13]. For instance, several frugivores require tree holes and fleshy-fruited trees for reproduction and feeding, respectively [14, 15]. Turacos are among the Afro-tropical montane forest specialist birds and play critical ecological roles mainly as seed dispersers [14]. Due to the rapid loss of forest cover and other factors in Africa, several turaco species are at risk of population decline. Moreover, *Tauraco ruspolii* of the Ethiopian endemic turaco and *T. bannermani* of West Africa are considered globally threatened [16]. Particularly, *Tauraco ruspolii* is restricted in a narrow range in southern Ethiopia and vulnerable to habitat degradation, illegal tree cutting, competition, and hybridization with the least concern *T. leucotis* [17, 18].

Research on the impact of climate change on avian species in Africa particularly in Ethiopia is untouched except [10] on the Ethiopian bush crow (*Zavattariornis stresemani*) and white-tailed swallow (*Hirundo megnensis*) and [19] on four highland birds. With this study, we employed one of the Species Distribution Models (SDMs), Maximum Entropy (MaxEnt, ), to predict the distribution and extent of suitable habitats of *T. ruspolii* and *T. leucotis* [20] under changing climates in Ethiopia. This model is the most popular and robust even with small occurrence points [21]. Thus, we aimed to determine the distribution and extent of suitable habitats and their influential predictors for the two turaco species under the changing climatic conditions. Furthermore, the study was aimed to calculate the suitable habitats overlap between the two turaco species.

## Materials and methods

### Species occurrence data

**Fig. 1:**
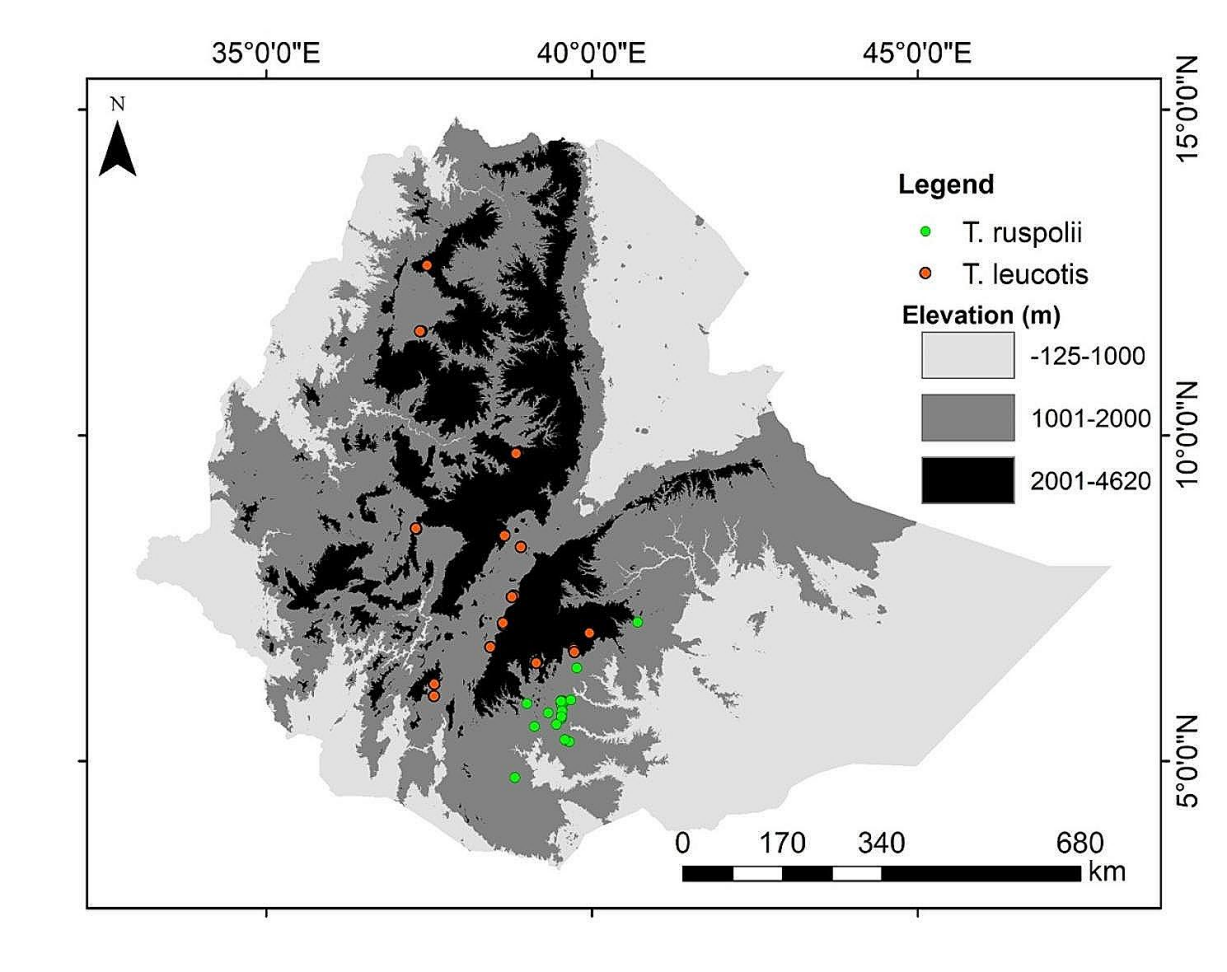
Occurrence points of the study species

The present study utilized the occurrence data of target species collected in Ethiopia (Fig. 1). Ethiopia is home to one of the richest and most unique assemblages of fauna and flora on the African continent [22]. In the country, about 863 bird species are believed to be recognized, of which 19 are endemic to the country alone and additionally 14 endemics shared with Eritrea [23]. The current studied turaco species are distributed in almost in common altitudinal range from 450 to 3600 m a. s. l [24].

We obtained 54 and 119 occurrence points for *T. ruspolii* and *T. leucotis*, respectively, from the Global Biodiversity Information Facility (GBIF, https://www.gbif.org) and previous published literature [25–28]. These occurrence points were spatially rarified with 1 km² spatial resolution using SDM toolbox V2.5 [29] to avoid spatial autocorrelations [30]. Thus, the retained 25 and 29 occurrence points of *T. ruspolii* and *T. leucotis*, respectively, were used for building habitat suitability models of the target taxa (Fig. 1; Table S1).

## Environmental variables

Habitat suitability of species and their spatial distribution depend on the cumulative interaction of various environmental variables [31]. In this study, 19 bioclimatic variables, topographic attributes, land use land covers as well as vegetation covers are considered.

The current bioclimatic data (i.e., an average of 1970 to 2000) were downloaded from WorldClim version 2.1 at a spatial resolution of 30s arc (1 km^2^) [32]. By assuming all current environmental variables will be unchanged, the future bioclimatic variables were also downloaded from the same source. The period of 2050s (2041–2060) and 2070s (2061–2080) with two shared socioeconomic pathways (i.e., the intermediate emission pathway-SSP4.5 and the worst-SSP8.5) developed by HadGEM2-Es global circulation models (GCMs) were used [32]. The topographic variables were derived from the Shuttle Radar Topography Mission Digital Elevation Model (SRTM-DEM) [33]. The Ethiopian vegetation types (http://landscapeportal.org/layers/geonode:veg.ethiopia) whereas land use land cover map was obtained from (https://cds.climate.copernicus.eu/) were also used. All of the environmental variables were processed using ArcGIS version 10.7 spatial analyst tools at 1 km^2^ resolution to have the same extent, projection and resolution [20].

To avoid multi-collinearity among environmental variables and increas model accuracy, we employed Pearson’s pair-wise correlation using DISMO package and then the Variance Inflation Factor (VIF) using USDM package [34] using R v4.2.2. For this, we first stacked 24 environmental variables and extracted their values at each of the occurrence points and additionally at randomly generated 10, 000 pseudo-absence points [35]. As a threshold, we used a correlation coefficient |r| ≤ 0.70 [30)] and VIF ≤ 10, and finally, we retained 13 of the same environmental variables for both species’ distribution and habitat suitability modeling (Fig. S1; Table 1).

**Table 1:** Selected variables and their contribution for model prediction after testing Pearson’s paired-wise correlation and Variance Inflation Factor (VIF)

Variables	Code	T. ruspolii		T. leucotis	
		VIF	Contribution (mean)	VIF	Contribution (mean)
Iso-thermality	Biol3	7.19	2.57	7.29	4.20
Temperature seasonality	Biol4	7.57	28.40	7.70	3.53
Temperature annual range	Biol7	2.11	0.17	2.13	0.00
Mean temperature of driest quarter	Biol9	3.42	0.00	3.36	39.77
Precipitation of wettest month	Biol13	6.96	2.97	6.81	2.30
Precipitation of driest month	Biol14	2.93	28.90	2.88	0.23
Precipitation seasonality	Biol15	3.01	4.20	3.01	2.80
Precipitation of warmest quarter	Biol18	2.34	10.30	2.31	0.37
Precipitation of coldest quarter	Biol19	3.76	3.93	3.65	2.90
Vegetation cover (categorical)	Vegetation	2.29	20.80	2.11	27.27
Land use land cover (categorical)	Lulc	2.44	0.67	2.21	23.17
Slope (categorical)	Slope	1.17	1.20	1.17	0.07
Aspect	Aspect	1.01	0.43	1.01	0.40

### Model setting and prediction

For both studied species, we used a similar model setting in Maximum Entropy (MaxEnt Version 3.4.4) [20]. The model also iterated 5000 times with 10 replications and used a default cross-validation run type. Regularization multiplier was set in three complex levels (labeled as 1Reg, 5Reg, and 8Reg) [36, 37]. The remaining settings were left as default. The predictive performance of the model was assessed using the Area Under Curve (AUC) of the Receiving Operator Characteristics (ROC) curve which provides a threshold-independent overall accuracy ranging between 0.5 and 1.0 [20]. Thus, models with AUC > 0.90 is considered to be high accuracy, 0.70 < AUC < 0.90 is good, 0.50 < AUC < 0.70 low accuracy and AUC ≤ 0.50 no better than randomness [38, 39].

We used two thresholds to classify the MaxEnt output maps into binary suitable / unsuitable: (1)10 Percentile Training Presence logistic threshold (10PTP) and (2) Maximum Test Sensitivity plus Specificity logistic threshold (MTSS). 10TP is explained as the predicted probability at 10% omission rate of the training data while MTSS is the probability of threshold at which the sum of fractions of correctly predicted presence and pseudo-absence points is the highest [40]. As a result, a total of six current binary maps (three regularization multiplier times two thresholds) and 24 binary maps for future projection (two future periods times two scenario times three regularization multiplier times two thresholds) were produced per species. Then, we applied ensemble approach for these binary maps and reclassified them into three habitat suitability classes based on agreements among the pixels [41; 42]: (1), pixels from less than 30% binary maps ( only one map for the current and up to three maps for each future projection period ) were considered as unsuitable; (2) between 30% and 60% binary maps (up to three for current and up to seven maps for each future projection period) were assumed to be uncertain and (3) above 60% (up to six for current and up to twelve for each future projection period) were considered to be suitable with high certainty.

Finally, species range size change between current prediction and each future projection period was employed to detect spatiotemporal change in habitat suitability for each species using the ArcMap version 10.7. Species range size change includes remain suitable, remain unsuitable, loss, gain, current range size, future range size and net species range size change. We also used UNION tool of the ArcMap to detect suitable habitat overlap between the two species [43]. The UNION tool provided three types of polygons: (1) the area of the polygons which has only T. *ruspolii* represent, (2) polygon which represents only the areas where *T. leucotis* present, (3) combined polygon which represents the areas of overlap for the two species. Then, we calculated the areas of these polygons and the area percentage of *T. ruspolii* with the suitable range of the *T. leucotis* and vice versa using the formula:

$$\text{Species 1}\left(\text{\%}\right)=\frac{\text{Area of overlap}}{\text{Area of species 1}} \times 100$$

## Results

### Variable importance and model performance

The average percent contribution of variables indicated that precipitation of the driest month (Biol14 = 28.9%), temperature seasonality (Biol4 = 28.4%), vegetation cover (20.8%), and precipitation of the warmest quarter (Biol18 = 10.8%) are the most determinant environmental variables for the habitat suitability prediction of *T. ruspolii* (Table 1). As the response curves revealed, *T. ruspolii* preferred habitats with Biol14 range from 10 to 20 mm (Fig. S2a). Its high habitat suitability (0.8) was also observed when the Biol4 (standard deviation x100) ranged from 50-100^o^C (Fig. S2b). During the warmest quarter, its habitat suitability increases until the precipitation (Biol18) reaches 300 mm then becomes stable (Fig. S2c). Desert and semi-desert scrubland, dry evergreen Afro-montane Forest and *Combretum-Terminalia* woodland are also the most preferable vegetation covers for this species.

On the other hand, the habitat suitability of *T. leucotis* is mainly influenced by the mean temperature of driest quarter (Biol9 = 39.77%), vegetation cover (27.27%) and land use land cover (23.17%) (Table 1). The non-linear response curve in (Fig. S3a) depicted that the habitat suitability of *T. leucotis* is negatively correlated with the mean temperature of the driest quarter (Biol9). Its most preferred vegetation cover is wide and ranges from Afro-alpine vegetation to *Combretum-Terminalia* woodland.

The AUC values of training and test datasets of the studied species are almost similar in the corresponding regularization multipliers. Since the AUC values of *T. ruspolii* are > 0.90, its model performance is found in high accuracy in all regularization multipliers and datasets (Table 2). The model also showed high performance accuracy for *T. leucotis* with the exception on the test dataset at 8Reg which failed with good accuracy (Table 2). The relation of regularizations and binary map thresholds indicated that the extent of predicted suitable habitat is reduced as regularization multipliers increase (i.e., model complexity decreases) in all thresholds for both species (Table 2). This implies that wide suitable habitat resulted in higher model complexity (1Reg) than at lower complexity (8Reg) regularization multiplier.

**Table 2:** Model performance and cut-off thresholds of binary maps for *Tauraco ruspolii* and *Tauraco leucotis*

Species	Regularization	AUC			Cut-off threshold	
		Training	Test	Diff	10PTP	MTSS
*T. ruspolii*	1Reg	1.00	0.99	0.01	0.35	0.33
	5Reg	0.99	0.99	0.00	0.58	0.57
	8Reg	0.99	0.98	0.01	0.66	0.63
*T. leucotis*	1Reg	0.97	0.92	0.05	0.14	0.24
	5Reg	0.90	0.93	-0.03	0.29	0.36
	8Reg	0.90	0.87	0.02	0.40	0.41

Diff: the difference between training and test AUC values

### Current and future habitat suitability

**Fig. 2:**
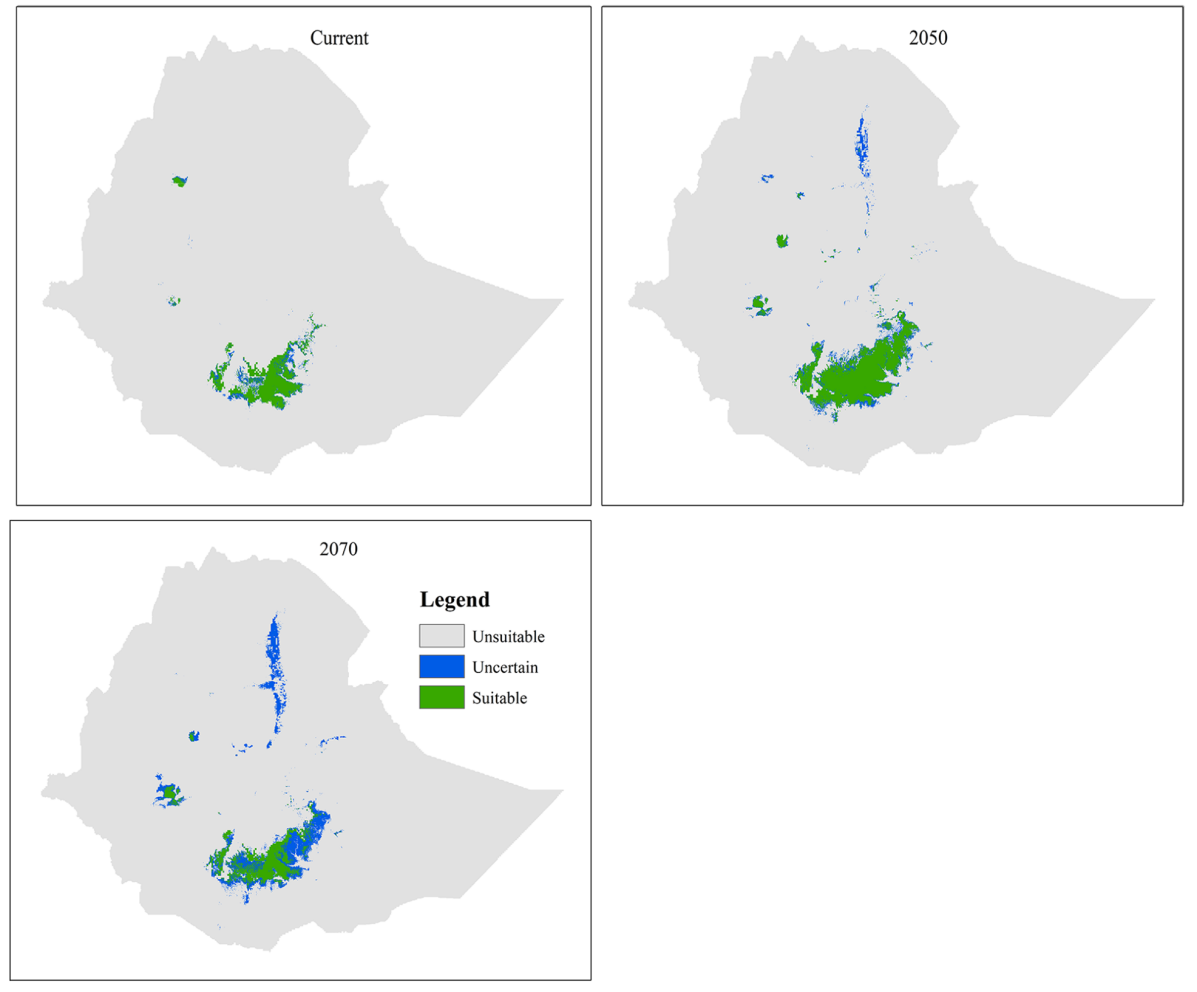
Ensemble habitat suitability of *T. ruspolii* from three class maps (suitable, uncertain and unsuitable) for the current and future climate conditions

The current predicted suitable habitat of *T. ruspolii* is mainly found in southern Ethiopia (relatively large extent), southwestern and northwestern Ethiopia (Fig. 2). Assembling of the future projection of *T. ruspolii* showed suitable habitat expansion with high certainty in both future periods (more pronounced in 2050) (Table 3). It is also more observed at southern Ethiopia.

Intersect of current with future climatic conditions indicated that *T. ruspolii* gain more than its current suitable habitat range (more than 100%) in both projected future periods (Table 3). Thus, the net habitat suitability change showed positive across the time series.

**Fig. 3:**
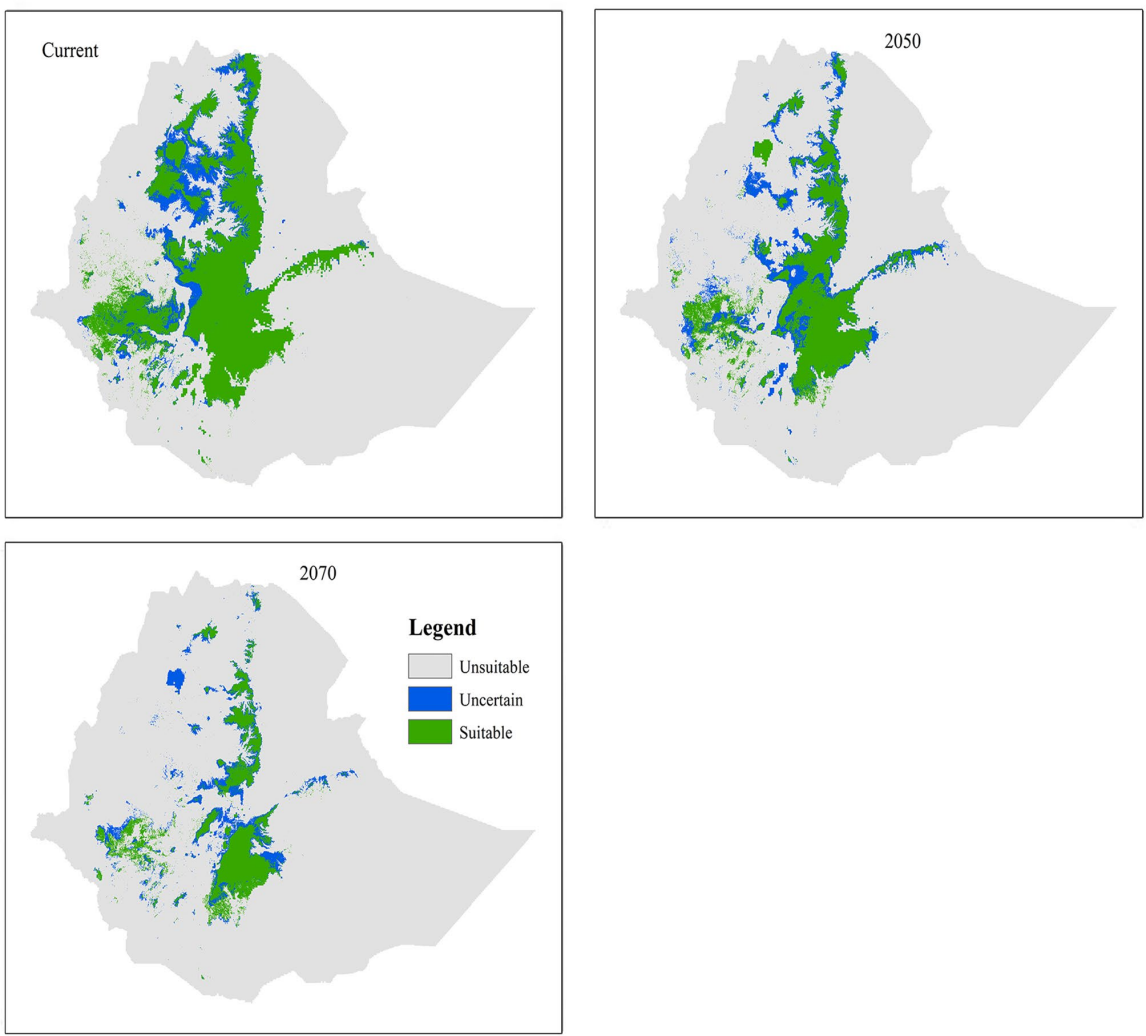
Ensemble habitat suitability of *T. leucotis* from three class maps (Suitable, Uncertain and Unsuitable) for the current and future climate conditions

Our model prediction has shown that *T. leucotis* has wide habitat suitability and distribution range relative to *T. ruspolii* under current and future climate conditions. The current predicted suitable habitat of *T. leucotis* is extended mainly from central Ethiopia towards south, southeastern, southwestern and northwestern Ethiopia with localities dominated by Afro-montane vegetation, dry evergreen forest, and *Acacia-Commiphora* woodland of the Rift Valley and *Combretum-Terminalia* woodland (Fig. 3).

**Fig. 4:**
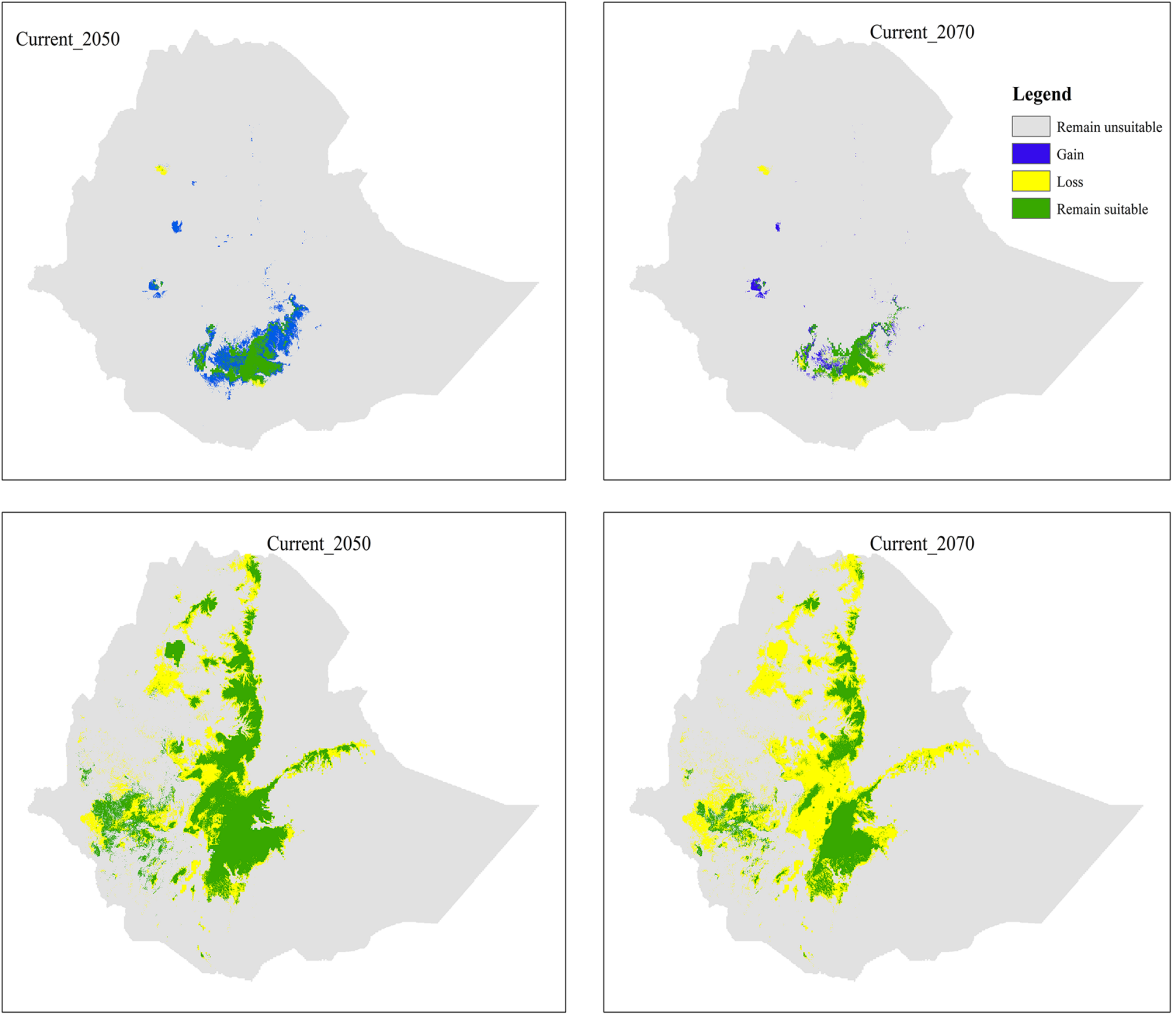
Intersect of the current tertiary maps with future climate conditions that showing projected habitat suitability change from current to future. The first row indicated the intersection of current with 2050s and 2070s of *T. ruspolii* whereas the second row indicated the intersection of current with 2050s and 2070s of *T. leucotis*

Unlike *T. ruspolii*, assembling the future projection of *T. leucotis* showed a reduction of suitable habitat range relative to the current climatic condition (Table 3). However, it would be still higher than the projection of *T. ruspolii*. The intersection of current with future climatic conditions also revealed this reality by showing a negative net change. *T. leucotis* lost more than 30.00% of its current suitable habitat range but gained less than 1.00% in both projection future periods (Table 3). The majority of its suitable habitats, particularly, in central Rift Valley, in Hararge highlands, and around Lake Tana are expected to be lost (Fig. 4).

**Table 3:** Temporal change in the extent of potential suitable habitats across the time range for *T. ruspolii* and *T. leucotis* in Ethiopia

Species	Intersection	Area (km²)					Change (%)		
		Remain Suitable	Gain	Loss	Current range size	Future projected Size	Gain	Loss	Net Change
*T. ruspolii*	Curr_2050	21903.20	41038.65	2735.99	24639.19	62941.85	167.00	11.00	156.00
	Curr_2070	22342.93	37418.64	2296.26	24639.19	59761.57	152.00	9.32	142.68
*T. leucotis*	Curr_2050	120510.92	413.72	83886.70	204397.62	120924.65	0.20	41.04	-40.84
	Curr_2070	63861.70	194.00	140534.00	204396.04	64055.7	0.09	68.76	-68.67

### Suitable habitat overlaps between species

**Fig. 5:**
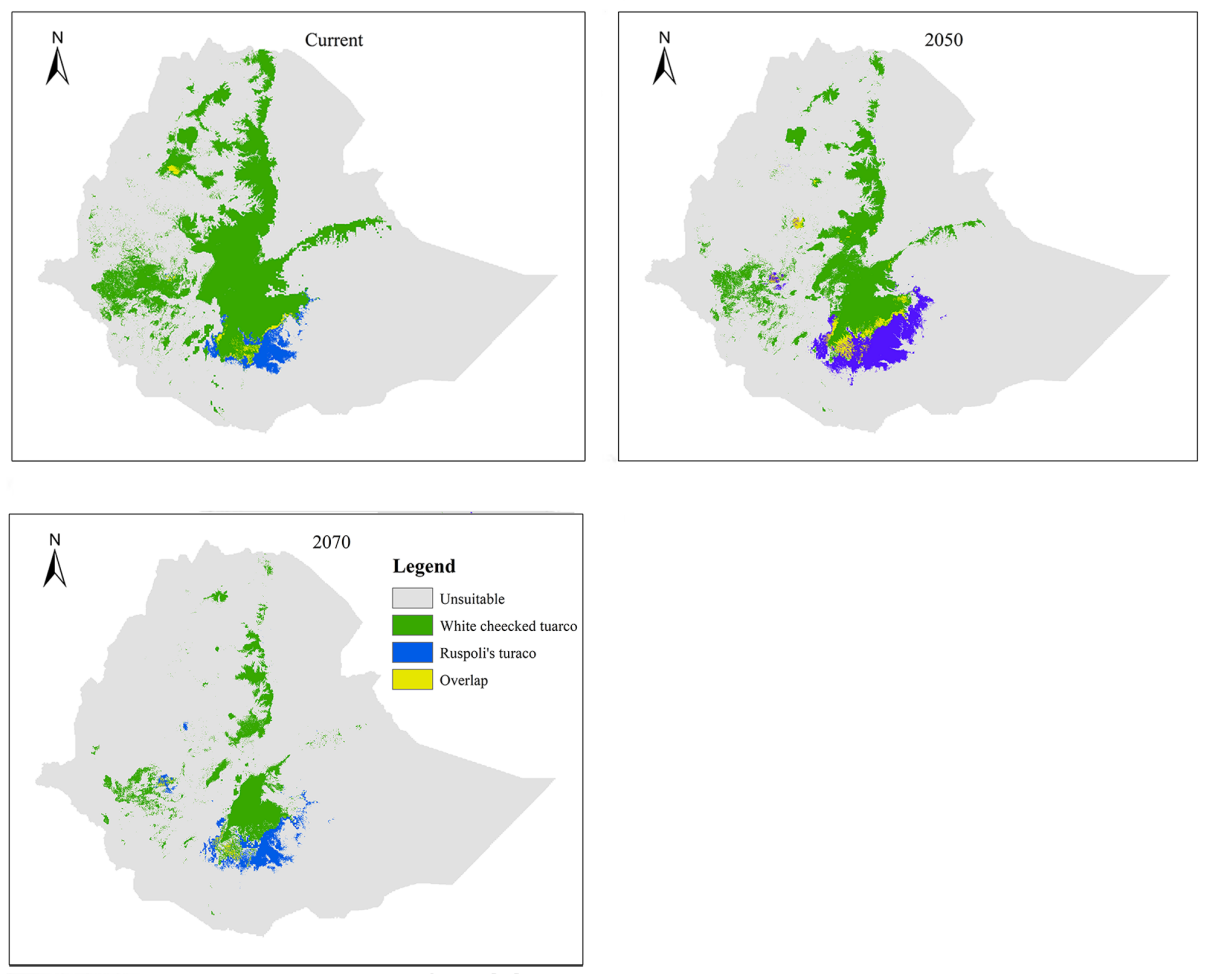
Suitable habitats overlap between *T. ruspolii* and *T. leucotis* across time series

Habitat suitability overlap between *T. ruspolii* and *T. leucotis* is observed in the projection periods with a slight shifting. It is found at the habitat margin of the respective species mainly in southern Ethiopia, northwestern Ethiopia (particularly, in Awi and East Gojjam), Western Ethiopia (East Wollega) and southeastern Ethiopia (Bale and Arsi areas) (Fig. 5).

Minimum habitat suitability overlaps between *T. ruspolii* and *T. leucotis* is observed in the 2070s whereas its maximum overlap is observed in 2050 (Table 4). As a general trend, the area percentage of *T*. *ruspolii* that overlaps with the range of *T. leucotis* is larger than the area percentage of *T. leucotis* that overlaps with the range of *T. ruspolii* in all periods (Table 4). However, a larger difference in area percentage overlap between these two species is observed in the current climate condition (15.89%).

**Table 4:** Area and percentage of suitable habitat overlap between *T. ruspolli* and *T. leucotis* under current and future climate conditions

Period	Area overlap (km²)	T. ruspolii (%)	T. leucotis (%)	Difference (%)
Current	3405.99	17.56	1.67	15.89
2050	7674.78	18.31	6.36	11.95
2070	1837.19	9.16	2.89	6.27

## Discussion

### Variable importance and model evaluation

Species Distribution Models are the most popular technique for assisting the management of specific species. The models may be constructed using various algorithms, predictors, and numbers of response variables and the confidence of their results depend on the goal and accuracy of the response variables. Among the bioclimatic variables, the model predicted that precipitation of the driest month (Biol14) and the warmest quarter (Biol18) affected the habitat suitability of *T. ruspolii*. This is because precipitation is linked to the richness of plant species and their primary productivity, thereby providing habitat requirements for their survival and success of reproduction [44]. For instance, Gwitira et al. [45] revealed that plant species richness increased as the precipitation of the warmest quarter increased up to 450 mm (which is not far from the result of this study i.e., 300 mm) in Southern Africa Savannah. Low precipitation in lowland areas is also a factor for the altitudinal shifting of lowland tropical birds to higher altitudes where the availability of resources is highest [9, 46]. However, extreme precipitation leads to the decline of reproduction due to flooding of nests, dying of broods and limitation of food provision for brood [47].

Vegetation cover is the common and most important factor for the habitat suitability of both *T. ruspolii* and *T. leucotis*. Our model predicted that desert and semi-desert scrubland (< 400 m a.s.l.) and dry evergreen Afro-montane forest (1500–3500 m a.s.l.) are suitable vegetation cover for *T. ruspolii* [48] whereas *T. leucotis* have shown wide vegetation cover range from Afro-alpine to *Combretum-Terminalia* woodland (above 900 m a.s.l.). The suitability of desert and semi-desert scrubland for *T. ruspolii* is unexpected compared to the fieldwork conducted from 1995 to 2003 [17, 25, 49] because they did not record the presence of both species in this vegetation type. However, range expansion of *T. ruspolii* to this vegetation type might occur during the wet season because this species is known to make localized seasonal movements [17]. Since turacos are frugivores, plant species that are found mainly in dry and moist evergreen Afro-montane forests are the main sources of fruits for these two turaco species [16, 50]. Borghesio [49] identified 10 plant species in dry evergreen Afro-montane forests as food resources, of which, *Ficus* species and two conifer species (*Juniperus procera* and *Podocarpus gracilior*) are the most preferred.

In this study, multiple maps were produced by applying different regularization multipliers (complexity levels). From these multiple maps, binary maps were also produced using different cut-off thresholds through an ensemble approach [41]. The use of different regularization multipliers has the advantage of enhancing the reliability of model performance and gives more confidence for taking conservation practice and management [42]. The AUC values of our model were greater than 0.90 for *T. ruspolii* and greater than and equal to 0.87 for *T. leucotis* (Table 2). These have shown that the model is found as high predictive performance for both species [38, 39]. However, the predictive performance can be influenced by different factors (variables) such as the extent of the study area and other species-related factors [51].

### Current and future habitat suitability

The result of MaxEnt prediction depicted that the current suitable habitat range of *T. ruspolii* is found in southern Ethiopia (relatively large extent), southwestern and northwestern Ethiopia with high fragmentation and covered about 24,639.19 km². This predicted suitable habitat range is less than the extent occurrence area of the species (26,800 km²) suggested by BirdLife International [18]. Out of this predicted suitable habitat range, a survey was conducted only on the southern Ethiopia (particularly around Negele Borena, Genale, Kibre Mengist, Shakiso and Arero) with the range of 8,000 km² [17]. The model indicated that northern Ethiopia (around Lake Tana) is a potentially suitable habitat range for *T. ruspolii*, but its presence has not been recorded yet. On the other side, *T. leucotis* has wide current suitable habitat ranges than *T. ruspolii* and can be extended from central Ethiopia towards south, southwestern, southeastern and northwestern Ethiopia. The model estimated the current area coverage of 204,397.62 km², which is less comparable with 1.1 million km² extent occurrence area of BirdLife International’s suggestion [52].

Assembling of the future projection of *T. ruspolii* indicated the expansion of suitable habitats in both 2050 and 2070 relative to the current climate condition (Table 3). As model prediction and previous field surveys [17] confirmed, this turaco species preferred dry evergreen Afro-montane forest which is its main food source. The expansion of suitable habitat in the future for this species might be due to two reasons: (1) dry evergreen Afro-montane forest covers wide range compared to other vegetation types of Ethiopia next to *Acacia-Commiphora* woodland [48]; (2) even though there is anthropogenic pressure in this vegetation type, there is also a potential dry forest management and conservation practices in different parts of the country including plantation development to be buffering for the natural forest which is managing by the government, controlling of overgrazing, traditional forest management practiced by different Ethnic groups like Gada system, a role model of sacred grooves, especially Ethiopian Orthodox Tewahedo Church [53–55].

In contrast to *T. ruspolii*, assembling of the future projection of *T. leucotis* in 2050 and 2070 indicated that loss is higher than gain, and thus, the net change will be negative (Table 3). The reason for this decline in the future might be due to the rise of the mean temperature of the driest quarter (Biol9) which in turn affects the availability of food resources. *T. leucotis* is also preferred in moist evergreen Afro-montane forests [17] where anthropogenic pressures (such as intensification of tea and coffee productivity, human settlement and dependency of the local people on the forest products) are severe and rapid [48, 56, 57]. Intensification of coffee productivity leads to the conversion of the natural coffee forest into fully plantation coffee causing significant plant diversity losses and collapse of forest structure [56, 58].

### Suitable habitat overlaps between the species

In this study, habitat suitability overlaps between *T. ruspolii* and *T. leucotis* were observed at the margin of their respective suitable habitats. Such overlap was also observed after 2001 during field survey. Before that, *T. ruspolii* preferred habitats of forest edge and *Acacia* woodland whereas *T. leucotis* mainly occurred in the moist (wetter) dense forests [17]. According to these authors, habitat degradation due to anthropogenic pressure is responsible for reducing the barriers between the two species. Wide suitable habitat overlap was predicted in the 2050s than the current climate condition and 2070s (Table 4). As a general trend, the area percentage of *T. ruspolii* that overlaps with the home range of *T. leucotis* is larger than the area percentage of *T. leucotis* that overlaps with the home range of *T. ruspolli* in all periods (Table 4). In other words, the model indicated that *T. ruspolii* will be expanded into the range of *T. leucotis*. This is in contrast to the field survey of 2003 [17]. Whatever the case, the overlapping of the two species’ habitat will lead to resource competition. Furthermore, hybridization between these two species was observed since 2001 [59] which indicates the presence of habitat overlap. Thus, the widespread hybridization at the overlap ranges leads to the risk of genetic erosion of the Nearly Threatened *T. ruspolii* [60] and which in turn leads to extinction despite the availability of suitable habitat in future climatic conditions.

## Conclusions

The study set out to determine the distribution and habitat suitability of *T. ruspolii* and *T. leucotis* using bioclimatic and non-bioclimatic factor. Model performance is found in high accuracy for *T. ruspolii* while good performance for *T. leucotis*. Precipitations of the driest month, temperature seasonality, and vegetation cover are the most contributor variables for the habitat suitability prediction of *T. ruspolii* while mean temperature of the driest quarter and vegetation cover for *T. leucotis*. The extent of both the current and future suitable habitat of *T. ruspolii* is less than that of *T. leucotis*. However, under future climate conditions, the extent of its suitable habitat is expected to be increased while this decreases for *T. leucotis*. Suitable habitat overlapping between the two species is also observed at the margin of their respective habitat types in current and future climate conditions. Therefore, understanding the distribution of current and future suitable habitats of these turaco species can provide valuable information to implement conservation practices for the species and the regions as well. A comprehensive survey for population assessment in highly suitable habitats is also fundamental to understanding the current conservation status of both species. Future research may also consider the application of numerous different models and their ensemble approach.

### Electronic supplementary material

Below is the link to the electronic supplementary material.

Supplementary Material 1

## Data Availability

No datasets were generated or analysed during the current study.
