# Supplementary material for: Distribution and extent of suitable habitats of Ruspoli’s Turaco (Tauraco ruspolii) and White-cheeked Turaco (Tauraco leucotis) under a changing climate in Ethiopia
Source: BMC Ecol Evol. 2024 Jun 21;24:83. doi: 10.1186/s12862-024-02245-y (PMC11191209; doi:10.1186/s12862-024-02245-y)
Supplement: Supplementary file 1 — Supplementary Material 1 [file 12862_2024_2245_MOESM1_ESM.docx]

**Supplementary Materials**

Distribution and extent of suitable habitats of Prince Ruspoli turaco (*Tauraco ruspolii)* and White-cheeked turaco (*Tauraco leucotis*) in times of climate change in Ethiopia

Mulatu Ayenew, Chala Adugna Kufa, Ahmed Seid Ahmed^3, 5^, Hailu Tilahun, Misganaw Tamirat, Mesele Yihune, Anagaw Atickem, Afework Bekele and Bezawork Afework Bogale

One Table and three figures

Table S1 Study species occurrence points

| *Tauraco ruspolii* | | *Tauraco leucotis* | |
| --- | --- | --- | --- |
| Longitude | Latitude | Longitude | latitude |
| 39.52672 | 5.910117 | 38.84825 | 9.712468 |
| 39.53677 | 5.931825 | 38.798 | 7.54 |
| 39.58169 | 5.916812 | 38.44216 | 6.750394 |
| 39.63351 | 5.307099 | 38.91023 | 8.285424 |
| 39.52451 | 5.886437 | 39.96128 | 6.96715 |
| 39.5546 | 5.91764 | 37.58035 | 6.181895 |
| 39.52715 | 5.931786 | 38.64153 | 7.081995 |
| 39.52816 | 5.853797 | 37.38443 | 11.59827 |
| 39.65227 | 5.300933 | 38.81514 | 9.732567 |
| 39.76517 | 6.431527 | 38.7899 | 7.528201 |
| 39.67646 | 5.939675 | 38.8093 | 7.534874 |
| 39.53957 | 5.789737 | 39.72254 | 6.70515 |
| 39.52924 | 5.650841 | 38.77044 | 7.518675 |
| 39.52881 | 5.719846 | 38.63555 | 7.102889 |
| 39.53278 | 5.671007 | 38.63824 | 7.09303 |
| 39.53862 | 5.771613 | 39.71471 | 6.744453 |
| 39.32682 | 5.742681 | 37.57413 | 5.999894 |
| 39.53068 | 5.685662 | 37.47131 | 12.60945 |
| 39.52449 | 5.922595 | 38.6348 | 7.121674 |
| 39.45465 | 5.565416 | 38.83568 | 9.721113 |
| 38.81667 | 4.75 | 39.72315 | 6.714918 |
| 39 | 5.883333 | 37.36176 | 11.59843 |
| 39.58333 | 5.333333 | 39.73076 | 6.674495 |
| 39.11667 | 5.533333 | 38.65645 | 8.473116 |
| 40.7 | 7.133333 | 38.65896 | 8.462312 |
|  |  | 39.16109 | 6.529362 |
|  |  | 39.14622 | 6.509047 |
|  |  | 37.30531 | 8.559749 |
|  |  | 37.29649 | 8.571432 |


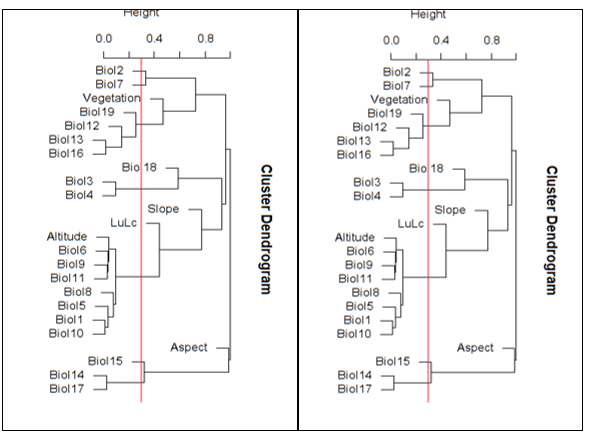


**Figure S1** Pairwise Pearson correlation of environmental variables for *Tauraco ruspolii* (left) and *Tauraco leucotis* (right). Redline shows a correlation coefficient r = |0.7|. Annual Mean Temperature (Biol1), Mean Diurnal Range (mean of monthly max temp - min temp; Biol2), Isothermality (Biol3), Temperature Seasonality (Biol4), Maximum Temperature of Warmest Month (Biol5), Minimum Temperature of Coldest Month (Biol6), Temperature Annual Range (Biol7), Mean Temperature of Wettest Quarter (Biol8), Mean Temperature of Driest Quarter (Biol9), Mean Temperature of Warmest Quarter (Biol10), Mean Temperature of Coldest Quarter (Biol11), Annual Precipitation (Biol12), Precipitation of Wettest Month (Bio13), Precipitation of Driest Month (Bio14), Precipitation Seasonality (Coefficient of Variation) (Bio15), Precipitation of Wettest Quarter (Bio16), Precipitation of Driest Quarter (Bio17), Precipitation of Warmest Quarter (Bio18), Precipitation of Coldest Quarter (Bio19), Slope, Aspect, Altitude, Land use land cover change (LuLc) and Vegetation.


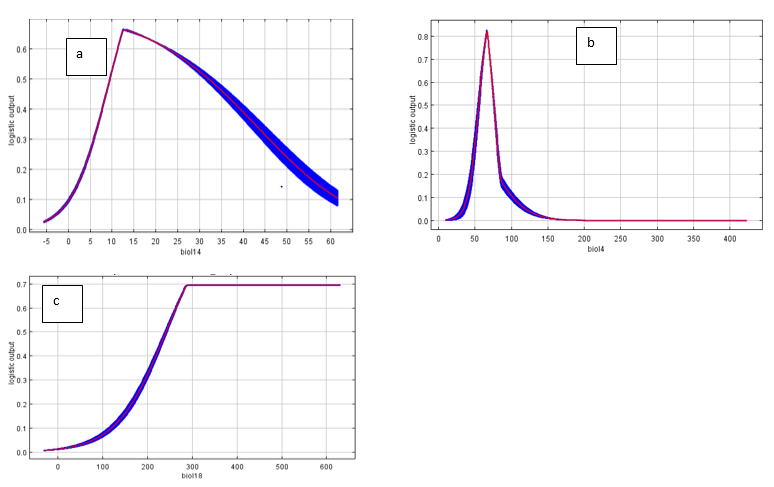


**Figure S2**. Response curve of the most important environmental variables on *T. ruspolii*: a) Precipitation of the driest month (Biol1), b) Temperature seasonality (Biol4), and c) Precipitation of the warmest quarter (Biol18)


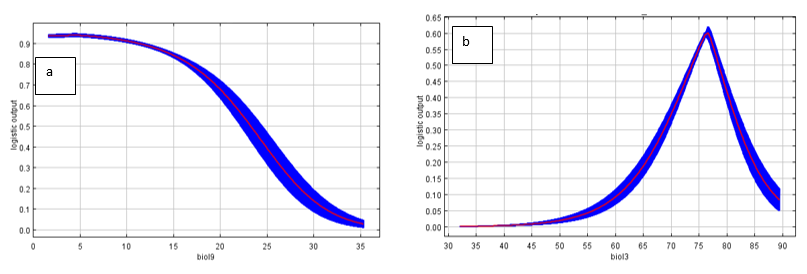


Figure S3: Response curve of the most important environmental variables on *T. leucotis*: a) Temperature of driest quarter (Biol9) and b) Isothermality (Biol3).
